# Characterization of the PIN Auxin Efflux Carrier Gene Family and Its Expression during Zygotic Embryogenesis in *Persea americana*

**DOI:** 10.3390/plants12122280

**Published:** 2023-06-12

**Authors:** Zurisadai Monroy-González, Miguel A. Uc-Chuc, Ana O. Quintana-Escobar, Fátima Duarte-Aké, Víctor M. Loyola-Vargas

**Affiliations:** 1 Centro de Investigación Científica de Yucatán, Unidad de Bioquímica y Biología Molecular de Plantas, Calle 43 No. 130 x 32 y 34, Chuburná de Hidalgo, Merida CP 97205, Yucatan, Mexico; zurisadai.monroy@estudiantes.cicy.mx (Z.M.-G.); ana.quintana@estudiantes.cicy.mx (A.O.Q.-E.); fapadake@hotmail.com (F.D.-A.); 2 Centro de Investigaciones Regionales Dr. Hideyo Noguchi, Avenida Itzáes, No. 490 x Calle 59, Col. Centro, Merida CP 97000, Yucatan, Mexico; ma.uc@outlook.com

**Keywords:** auxin, *Persea americana*, PIN, transport, zygotic embryogenesis

## Abstract

Auxins are responsible for a large part of the plant development process. To exert their action, they must move throughout the plant and from cell to cell, which is why plants have developed complex transport systems for indole-3-acetic acid (IAA). These transporters involve proteins that transport IAA into cells, transporters that move IAA to or from different organelles, mainly the endoplasmic reticulum, and transporters that move IAA out of the cell. This research determined that *Persea americana* has 12 PIN transporters in its genome. The twelve transporters are expressed during different stages of development in *P. americana* zygotic embryos. Using different bioinformatics tools, we determined the type of transporter of each of the *P. americana* PIN proteins and their structure and possible location in the cell. We also predict the potential phosphorylation sites for each of the twelve-PIN proteins. The data show the presence of highly conserved sites for phosphorylation and those sites involved in the interaction with the IAA.

## 1. Introduction

Plant development is regulated by multiple factors, including plant growth regulators (PGRs), mainly auxins and cytokinins. Indole-3-acetic acid (IAA), the main auxin form, has a pivotal role in almost all plant development processes [1,2,3]. This ability to influence all the corners of the plant is due to the possibility of regulating its cell-to-cell transport, local synthesis, inactivation, and conjugation [4].

IAA is synthesized in young leaves, cotyledons, fruits, seeds, developing flowers, and roots [2,5,6]. Afterward, auxin is transported throughout plants by a fast, non-polar transport through the phloem and by diffusion or a slow cell-to-cell polar auxin transport (PAT) [7]. PAT is essential in various auxin-mediated developmental processes, due to the fact that it is needed for the formation of auxin gradients in plants [8,9].

PAT is mediated by the influx and efflux carriers, including AUXIN RESISTANT 1/LIKE AUX1 influx carriers (AUX1/LAX) [10,11], ATP-binding cassette (ABC) subfamily B [12,13,14], PIN FORMED proteins (PIN) [15,16,17], PIN-Like transporters (PILS) [18,19], nitrate transporter 1.1 (NRT1.1) [20] and WALLS ARE THIN 1 (WAT1) [21]. Among the transporters mentioned above, the PIN efflux carriers are the primary transporters that control the PAT. For a recent reviews on auxin transporters, see Marhava et al. [22], Hammes et al. [9], Vosolsobê et al. [23], and Geisler [1].

The auxin transporters can be found in the entire green lineage (*Viridiplantae*) [23,24]. This abundance of auxin transporters suggests they have a critical role in plant development. This function can occur from its presence in cell membranes to directing the polar traffic of auxins over great distances; from transporters in intracellular membranes, primarily involved in maintaining auxin homeostasis within the cell and creating the auxin accumulation that leads to the cell’s response to auxin, to transporters capable of moving other molecules besides auxin under specific physiological conditions [9].

PIN homologs have been identified in more than 31 plant species [16], including *Oryza sativa* [25], *Solanum lycopersicum* [26], *Glycine max* [27], *Populus trichocarpa* [28], *Triticum aestivum* [29], *Coffea spp* [30], *Mikania micrantha* [31], *Panax ginseng* [32], *Medicago truncatula* [33], *Zea mays* [34], *Gossypium barbadense* [35], and *Olea europaea* [36].

PIN proteins are integral membrane proteins. The structure of PIN proteins has ten highly conserved transmembrane domains (TMD) and a divergent central hydrophilic loop (HL) located in the cytoplasm [17,24,37]. *Arabidopsis thaliana* has eight members of the PIN protein family, classified into two groups based on the size of the central HL: long PIN proteins (canonical), AtPIN1-AtPIN4, AtPIN6, and AtPIN7; and short PIN proteins (non-canonical) AtPIN5 and AtPIN8 [15]. Long PINs are localized at the plasma membrane (PM), whereas short PINs are localized at the endoplasmic reticulum (ER) [15,16]. AtPIN6 is a particular case. It has a dual localization at the PM and the ER [38].

The number of PIN genes varies widely among higher plants. It can be as small as in *Marchantia polymorpha* (4) [24] and *Carica papaya* (6) [39], intermediate, as in *A. thaliana* and *A. lyrata* (8) [15,24], *Vitis vinifera* (8) [15,40] *Capsicum annuum* (10) [41], *Solanum lycopersicum* 10 [26], *Citrus lanatus* (11) [42], and *Oryza sativa* (12) [43], or as high as in *Zea mays* (15) [34,44] *Gossypium hirsutum* (17) [45,46], *Nicotiana tabacum* (20) [47] or *Glycine max* and *Phyllostachys edulis* (23) [27,48,49]. The number of PIN genes even varies within the same genus, as in the case of *Gossypium*; *G. arboreum* (12) [45], *G. hirsutum* (17) [45,46], and *G. raimondii* (19) [45].

The abundance of PIN transporters has allowed plants to allocate each to a different function, allowing finer control of auxin transport. In maize, it was observed that ZmPIN1 plays a fundamental role in embryogenesis and endosperm development [44,50]. In cotton, PIN proteins are essential for fiber development [45], and PIN1-3 and PIN2 are required for root development [46]. In *O. sativa*, OsPIN1 is involved in adventitious root emergence and tillering [51] and OsPIN1b regulates root gravitropism [52] and leaf inclination [53]. In *A. thaliana*, PIN transporters participate in the response to heavy metal stress [11,54,55]. AtPIN4 is involved in generating a sink for auxin at the apical resting center [56]. In the embryogenesis process, PIN1, PIN3, PIN4, and PIN7 are required for apical–basal axis formation of the embryo, and auxin gradient establishment [57,58,59,60]. In A. thaliana, the PIN transporters (particularly PIN3) also participate in hypocotyl hook development during skotomorphogenesis [61].

The ATP-binding cassette (ABC) of membrane transporters is one of the largest and most ancient known protein superfamilies found in living organisms [62]. This family of proteins is involved in the transport of plant growth regulators, e.g., the OsABCG18 plays an important role in the transport of cytokinins [63], and the Arabidopsis MDR/PGP transporter AtPGP1 and AtABCB4 are involved in the efflux of auxin [64,65]. ABC transporters also participate in the mobilization of pigments [66], toxic chemicals [67], and the secretion of chemicals by the roots [68].

PIN genes have been identified in over thirty plant species [16]; however, PIN genes have not been characterized in *Persea americana*. This study analyzed the phylogenetic relationship, gene structure, conserved motif, and expression profile in silico in avocado fruit. To our knowledge, this is the first report of the PIN gene family in the avocado genome. These results will contribute to a better understanding of the characteristics of PINs and provide the basis for future functional characterization of the PIN gene family in the avocado and their role in embryo development.

## 2. Results

### 2.1. Identification of PIN Gene Family in Avocado

Twelve *PaPIN* genes were identified in the avocado genome. The nucleotide sequence length ranged from 1074 to 1983 bp (base pairs). The lengths of the corresponding proteins ranged from 357 to 660 amino acids, and they possessed 39.16 kDa to 71.91 kDa molecular masses and pI (isoelectric point) values of 6.95 to 9.45. The calculated average of the hydropathy index (GRAVY) values of avocado PaPINs varied from 0.086 to 0.722 (Table 1). The subcellular localization prediction of PaPIN proteins suggests that most PINs of the avocado are located in the plasma membrane, except PaPIN1a and PaPIN5, which are located in the chloroplast and vacuole, respectively. Between eight and nine transmembrane helices were predicted, except for PaPIN2b, which has five transmembrane helices.

### 2.2. Phylogenetic Analysis of PaPIN Family

**Figure 1:**
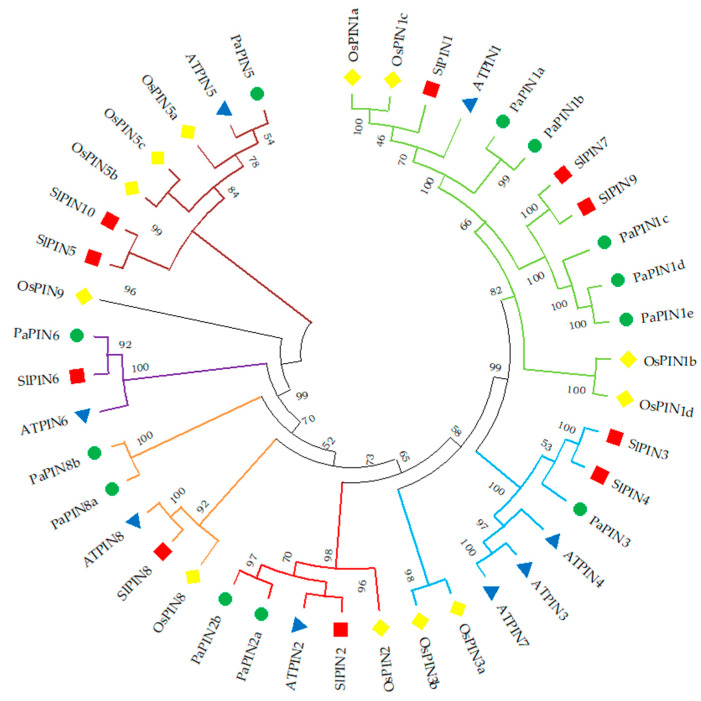
Phylogenetic analysis of PIN among four plant species. At: *Arabidopsis thaliana*; Os: *Oryza_sativa*; Sl: *Solanum lycopersicum*; Pa: *Persea americana*. The phylogenetic tree was constructed with the MEGA11 version software, using the neighbor joining method. The PIN protein clusters are indicated in different colors.

A phylogenetic tree was constructed using the sequences of the protein family PINs from *A. thaliana*, *O. sativa* and *S. lycopersicum*, and *P. americana*. This study used 24 amino acid sequences (Figure 1). The PINs proteins of *P. americana* were named based on their homologous relationship with *A. thaliana*. The PIN family of *P. americana* includes the *PaPIN1*, *PaPIN2*, *PaPIN3*, *PaPIN5*, *PaPIN6*, and *PaPIN8* genes. In addition, the PIN1 of avocado has five paralog genes (*PaPIN1a*, *PaPIN1b*, *PaPIN1c*, *PaPIN1*d, and *PaPIN1e*), whereas PIN2 and PIN8 had two paralogs genes (*PaPIN2a* and *PaPIN2b*) and (*PaPIN8*a and *PaPIN8b*) respectively; in total, twelve PIN genes were identified in avocado.

PIN proteins are divided into six clusters (Figure 1). Group I includes the PIN1 proteins from *A. thaliana*, rice, and avocado PIN7 and PIN9 from tomato. Group II is made up of PIN3, PIN4, and PIN7. In contrast, group III comprises PIN2a and PIN2b of avocado, rice, tomato, and Arabidopsis. Two clades are observed in group VI; the first includes Arabidopsis PIN5, rice, and avocado, and the second includes tomato PIN5 and PIN10. Group V is the smallest group, represented by PIN6 from *Arabidopsis*, *Solanum*, and *Persea*. Meanwhile, two PIN8 paralogs from avocado, along with rice, tomato, and Arabidopsis, are observed in group IV.

Interestingly, we found 92% homology between the PaPIN6 proteins with SlPIN6, while PaPIN5-AtPIN5 and PaPIN3-SlPIN3 only had 54 and 53% homology, respectively. These data indicate a clear phylogenetic relationship between the PaPIN proteins with the sequences of the PIN proteins of *A. thaliana*, *O. sativa*, and *S. lycopersicum*.

### 2.3. Gene Structure Analysis, Transmembrane Region Prediction and Conserved Motifs of PIN Genes in Avocado

**Figure 2:**
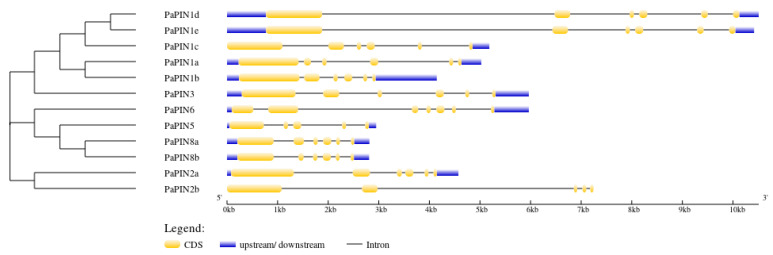
Exon–intron structure analysis of PINs in *Persea americana* using GSDS (http://gsds.gao-lab.org/ (accessed on 17 October 2022)). The yellow boxes denote the CDS (Coding Sequence), the blue lines show the UTR (Untranslated Regions), and the black lines represent the intron.

Gene structure analysis was performed by comparing the PIN genes’ coding and genomic sequences from *P. americana*. The results showed that most PINs genes in *P. americana* contain six exons, except for *PaPIN2b* and *PaPIN5*, and *PaPIN*6, with five and seven exons, respectively. All *PaPIN* genes had two untranslated regions (UTRs), except *PaPIN1c*, which did not have 5’ UTR, whereas *PaPIN2b* did not have UTRs. The largest intron was observed in *PaPIN1d* and *PaPIN1e*. The number of introns ranged from four to six (Figure 2).

**Figure 3:**
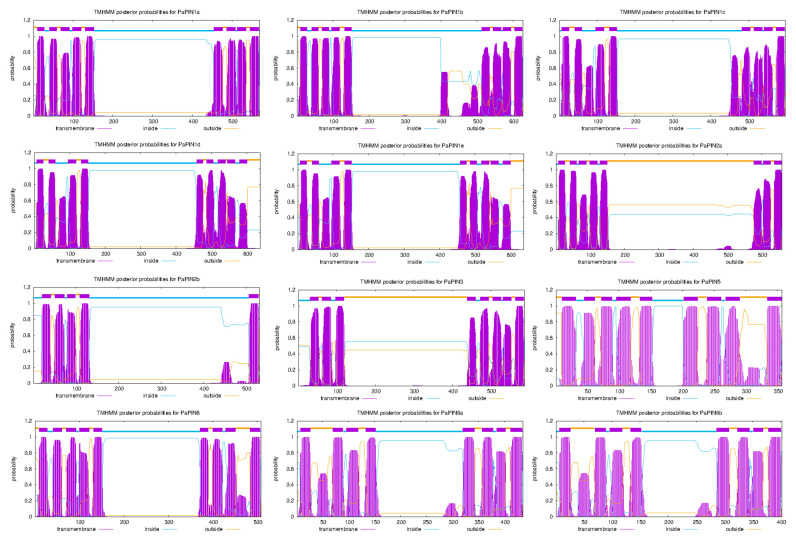
Prediction of transmembrane regions for *P. americana* PaPIN proteins using TMHHM v.2.0 M software (transmembrane prediction using hidden Markov models). The predicted transmembrane domains are shown in purple, regions on the cytoplasmic side are indicated in blue, and regions outside are marked in orange. The genes PaPIN1d and PaPIN1e have the same number of amino acids (368) and are identical copies, giving the exact prediction across the membrane.

The transmembrane helices of PaPIN proteins were predicted using TMHHM v.2.0. The number of transmembrane helices ranged from eight to nine, except for PaPIN2b, with five helices. All PaPINs proteins showed a similar structure comprised of conserved transmembrane domains at the N-terminal and C-terminal region and a central hydrophilic loop localized in the cytoplasm (Figure 3). Based on the results obtained, the PINs of avocado were classified into long PaPINs, integrating PaPIN1a, PaPIN1b, PaPIN1c, PaPIN1d, PaPIN1e, PaPIN2a, PaPIN2b, PaPIN3, and PaPIN6; and short PaPINs, composed of PaPIN5, PaPIN8a and PaPIN8b. Furthermore, the average length of the central hydrophilic loop was 341 amino acids for long PaPINs. On the other hand, for short PaPINs, the length of the central hydrophilic loop was 50, 168, and 134 amino acids for PaPIN5, PaPaPIN8a, and PaPIN8b, respectively.

**Figure 4:**
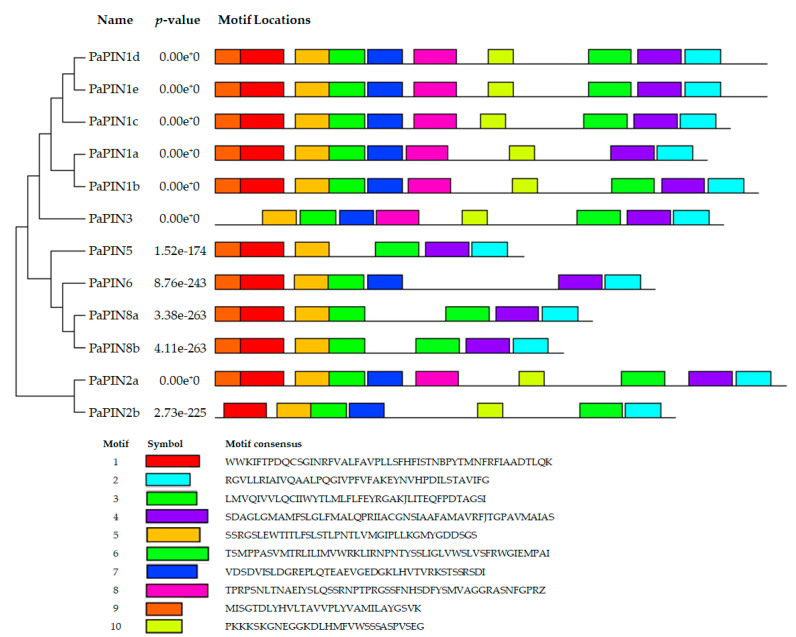
Conserved motif analysis of PIN proteins of *P. americana*. Different color boxes represent motifs.

The conserved motifs of the PIN proteins in avocado were identified using Multiple EM for Motif Elicitation (MEME). The motifs of the PaPIN1b,c,d,e, and PaPIN2a proteins display the ten characteristic motifs found in other species [49]. PaPIN1a only has nine motifs. The PaPIN3 protein has eight motifs, and the PaPIN6, PaPIN8a,b, and PaPIN2b proteins have seven motifs. The PaPIN5 protein has only six motifs. In addition, motifs 1, 3, 5, and 9 were conserved at the N-terminus region, and motifs 2, 4, and 6 were conserved at the C-terminal region (Figure 4).

### 2.4. Building of 3D Structures, Molecular Modeling and Multiple-Sequence Alignment of PaPIN Proteins from P. americana

**Figure 5:**
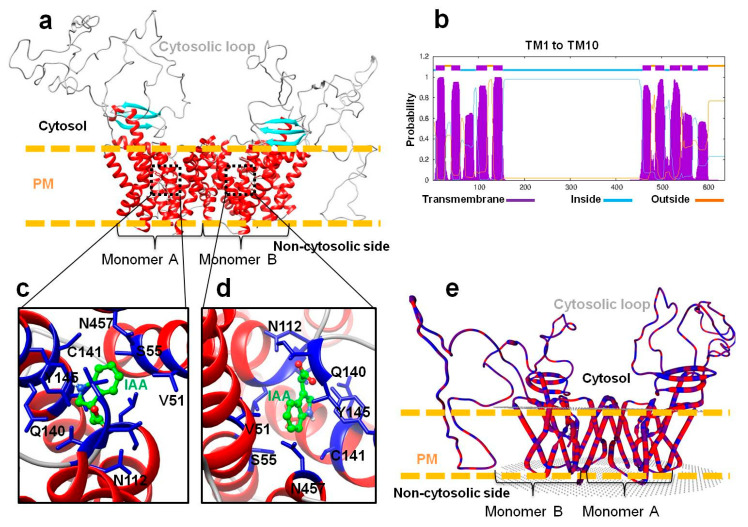
Modeling and prediction of transmembrane segments of the PaPIN1e protein from *P. americana*. (**a**) Homodimeric conformation of the auxin transport protein PaPIN1e. The transmembrane domain is shown in red, the beta-sheets in cyan, and the cytosolic loops in grey. (**b**) Prediction of transmembrane (TM) segments. (**c**,**d**) IAA binding in the pocket of PaPIN1e. IAA is shown in green spheres. Residues that possibly interact with IAA are shown in blue and highlighted in black letters. (**e**) PaPIN1e hydrophobicity is shown in rope view. The regions in red are hydrophobic, and in blue they are less hydrophobic. Red regions indicate aliphatic residues.

**Figure 6:**
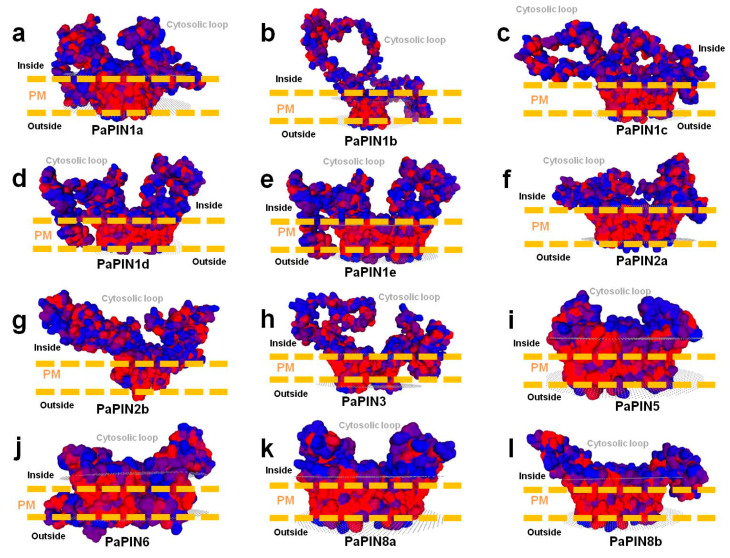
Surface view of the electrostatic potential of twelve members of the family of auxin transport proteins PaPINs from *P. americana* (**a**–**l**). The regions in red are hydrophobic, and the regions in blue are less hydrophobic. PM, the plasma membrane, is shown in discontinuous yellow lines. Cytosolic regions are labeled in blue. All the proteins modeled in this study contain a hydrophobic domain with 10 TMs.

In the present study, we built 3D structures of twelve members of the PaPIN family of *P. americana* (Figure 5a,c,d and Figure 6a–l). The 3D structures of the PaPIN1b, PaPIN1d, and PaPIN1e proteins were constructed by homology using the 7XXB PDB template code, while the rest of the PaPINs were modeled with the 7WKS PDB template. The predicted structure forms a homodimer, with each monomer (A and B) divided into a transport domain with a clearly defined IAA auxin binding site (Figure 5a,c,d). The canonical PaPIN structures are membrane transporters, presenting polar localization in the plasma membrane of the plant cell (Figure 5a and Figure 6a–l). This polar condition provides IAA transport directionality from cell to cell [69].

**Figure 7:**
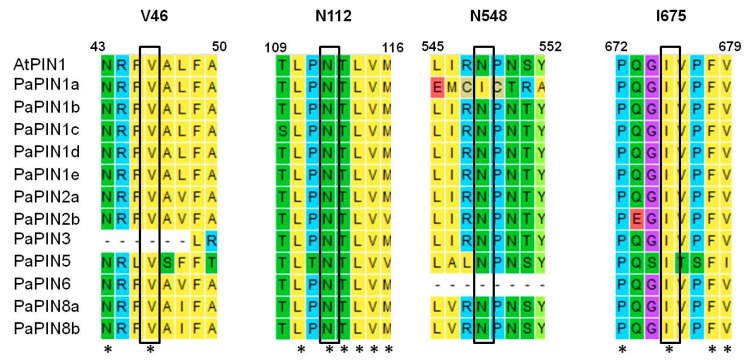
Multiple alignment of PaPIN proteins from *P. americana*. AtPIN1 was used as a reference protein. Colors indicate conserved amino acids, however this is not the case in all sequences. While those marked with an asterisk indicate highly conserved amino acids at a specific point in the sequences. IAA interacting residues are shown above (V, valine; N, asparagine; I, isoleucine) and in black boxes. Asterisks show conserved residues.

The predictions of the 3D structures of the members of the PaPIN family showed the transmembrane domain of ten transmembrane segments (TM1 to TM10) typical of these proteins (Figure 3 and Figure 5a,b). The N and C termini are located extracellularly, and the hydrophobic region is transmembrane (Figure 5a,b), as previously reported in *Arabidopsis thaliana* [6,15]. The prediction of the IAA binding site in the pocket of PaPIN1e showed the interaction between the IAA molecule and residues V51, S55, N112, Q140, C141, Y145, and N457 in the hydrophobic region of both homodimers (Figure 5c,d). The structure of AtPIN1 was recently elucidated by cryo-electron microscopy (Cryo-EM), in which the IAA molecule is coordinated through hydrogen bonds and hydrophobic interactions [6]. In addition, a water molecule forms hydrogen bonds to bridge the amino group of the imidazole ring of the IAA with residue N112 in AtPIN1 [6]. Residues interacting with IAA have been reported to be V46, N112, N548, and I675 [6]. These residues are highly conserved in the twelve PaPIN proteins modeled in this study (Figure 7). Additionally, it has been documented that the region that corresponds to the cytosolic loop or hydrophilic loop (Figure 5a and Figure 6a–l) has target sites for kinases such as PINOID for the phosphorylation of serine or threonine residues; this action is crucial for the polar location of the PINs in the plasma membrane [70,71,72].

All the 3D structures predicted in this study showed hydrophobic regions comprising 10 TMs, and presented the characteristic hydrophilic loop of auxin transport proteins (Figure 3 and Figure 6a–l). In addition to the IAA interaction residues (V46, N112, N548, and I675), the topology of the twelve PaPINs modeled in this work is similar to the structures of AtPIN1 recently reported [6]. This study’s theoretical data from molecular modeling indicate that PaPINs are structurally conserved and might exhibit auxin transport activity in *P. americana*.

### 2.5. Prediction of the Phosphorylation Sites in the PaPIN Proteins

**Figure 8:**
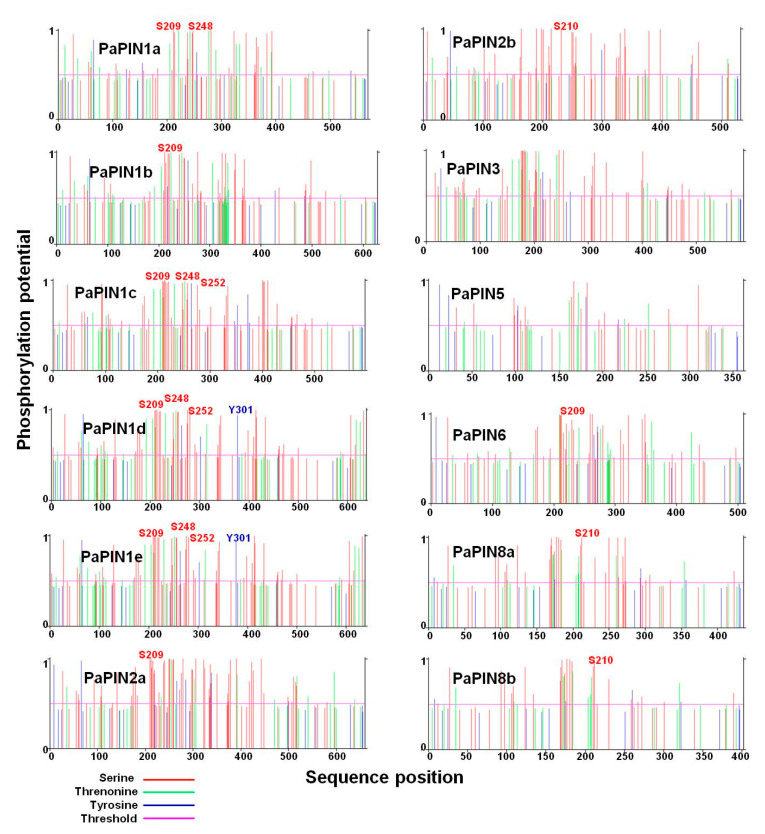
Theoretical prediction of phosphosites in the auxin transport proteins PaPINs of *P. americana*. The phosphorylated residues are serine, in red, threonine, in green, and tyrosine, in blue. Peaks above the threshold (horizontal magenta line) indicate predicted phosphorylation sites. Phosphorylated conserved residues are numbered at the position predicted in each PaPIN protein and indicated by the letters S (red) and Y (blue). The ordinate axis corresponds to the predicted phosphorylation potential, and the abscissa axis is the position of the sequences of the residues in the PaPIN proteins.

The amino acid sequences of the 12 members of the PaPIN protein family were used to predict the phosphorylation sites (phosphosites). Theoretical results showed that the twelve-member PaPINs undergo post-translational modification by phosphorylation (Figure 8). The predictions showed that serine (S), threonine (T), and tyrosine (Y) were the phosphorylated residues, with serine being the predominant residue modified by phosphorylation. The predicted phosphorylation peaks were located between the amino acids 200 and 400. In addition, it was possible to predict different phosphosites in the N-terminal region of the PaPIN proteins. This region comprises the hydrophilic loop of PaPIN proteins (Figure 8). Phosphorylation at the S209 residue is conserved in the PIN1a, PIN1b, PIN1c, PIN1e, PIN2a, and PIN6 proteins. We also found that the S210 residue is phosphorylated in the PaPIN2b, PaPIN3, and PaPIN8a-b proteins, while S248 phosphorylation was only found in the PaPIN1a, PaPIN1c, PaPIN1d, and PaPIN1e proteins. On the other hand, we found residues Y302 and T252 phosphorylated in the PaPIN1d, PaPIN1e, and PaPIN1c, and the PaPIN1d, and PaPIN1e proteins, respectively.

### 2.6. Differential Expression Analysis of PINs in Zygotic Embryo in Avocado

**Figure 9:**
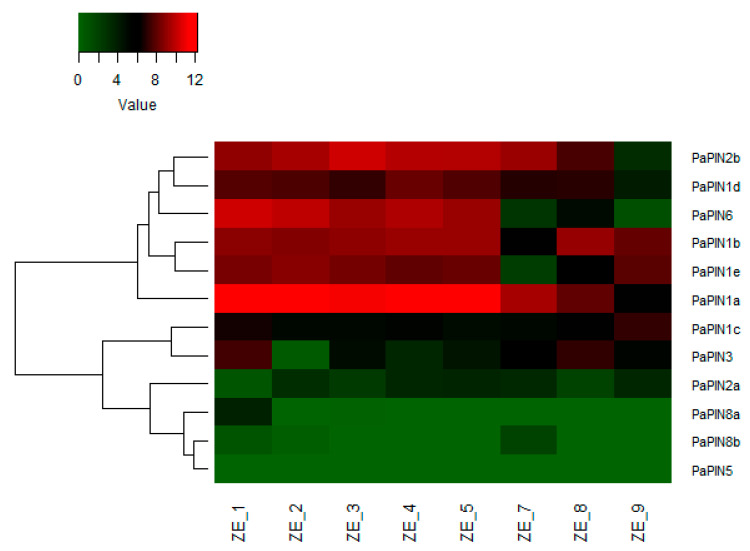
Expression profile of PIN-FORMED genes involved in the transport of IAA during the development of the zygotic embryo in P. americana cv. Hass. The green color denotes higher expression levels, while the red represents the lower ones. ZE: zygotic embryo; numbers 1 to 9: fruit sizes (cm) used to extract zygotic embryos of avocado.

To determine the level of expression of the PIN genes throughout the formation of the zygotic embryo, we analyzed a transcriptome carried out with zygotic embryos from eight fruit sizes (1 cm: ZE_1; 2 cm: ZE_2; 3 cm: ZE_3; 4 cm ZE_4; 5 cm: ZE_5; 7 cm: ZE_7; 8 cm: ZE_8 and 9 cm: ZE_9). It was determined that 12 PIN genes are expressed in *P. americana* zygotic embryos (Figure 9 and Table 1), which are homologous with *A. thaliana*, *O. sativa*, and *S. lycopersicum* (Figure 1).

Based on their expression profile, the avocado *PIN* genes were clustered into two groups; the first group showed high expression during the early stages of embryo development. In contrast, the second group showed low-profile expression throughout the embryo’s development. Six genes, including *PaPIN2b*, *PaPIN1d*, *PaPIN6*, *PaPIN1b*, *PaPIN1e*, and *PaPIN1*a, had high expression levels in ZE_1 to ZE_5, which, of the *PaPIN1a*, showed a higher expression level. *PaPIN1a* and *PaPIN2b* showed high expression levels from ZE_1 to ZE_8. However, the expression level of *PaPIN2b* was lower in ZE_9 compared to *PaPIN1a*. The second group included *PaPIN1c*, *PaPIN3*, *PaPIN2*a, *PaPIN8a*, *PaPIN8*b and *PaPIN5* genes. Compared to *PaPIN1c*, *PaPIN3* y *PaPIN2b*, the expression of *PaPIN2a*, *PaPIN8a*, *PaPIN8b* y *PaPIN5* was lower throughout the development of the embryo. In addition, *PaPIN1c* did not show differences in expression in all conditions evaluated, except in ZE_9. In contrast, *PIN3* showed a high expression level in ZE_1 and ZE_8.

These results suggested that *PaPIN1a*, *PaPIN2b*, *PaPIN1d*, *PaPIN6*, *PaPIN1*b, and *PaPIN1e* are required in the early stages of embryo development of the avocado.

## 3. Discussion

Auxin, in its most abundant form IAA, is perhaps the molecule involved in almost all aspects of plant life [69,73]. In particular, IAA is vital for the coordinated development of all phases of somatic embryos [74,75,76,77]. IAA requires local biosynthesis [78] and PAT from cell to cell [15,79] to perform its function. PAT is carried out by members of the PIN-FORMED family [15] and members of the ABC transporter family [12,80,81].

Avocado somatic embryogenesis (SE) still has deficiencies in its use in research and its possible applications [82,83]. Data from our laboratory during the SE process (not shown) show an unusual behavior in the content of auxin during the SE induction process. Our laboratory rationalized that it is possible that the problem of low SE efficiency in avocado and its poor regeneration is due to how the IAA is distributed during the process. IAA must accumulate in a particular cell type in the developing embryo [84]. For this reason, we searched for the PIN genes in the recently sequenced avocado genome [85]. We wanted to explore whether this characterization during the zygotic embryogenesis process could help us solve the SE problem in the avocado.

Auxin transporters are present from algae [40,86] to higher plants [23]. Despite their importance, we know little about PIN carriers. The most significant and best knowledge of this transporter type comes from *A. thaliana* [87,88], and little is known beyond rice and Arabidopsis.

We found twelve *PaPIN* genes in the avocado genome (Table 1; Figure 1). *Solanum lycopersicum* has ten *SlPIN* genes [26], the model species *A. thaliana* has eight *AtPIN* genes (AtPIN1 to AtPIN8) [87,88] and in the monocot *Oryza sativa* there are twelve [25]. What is very important is the distribution of the different *PIN* genes in the avocado. These genes are distributed as follows: *PaPIN1*, *PaPIN2*, *PaPIN3*, *PaPIN5*, *PaPIN6*, and *PaPIN8* genes. In addition, *PaPIN1* has five paralog genes (*PaPIN1a*, *PaPIN1b*, *PaPIN1c*, *PaPIN1*d, and *PaPIN1e*), whereas *PaPIN2* and *PaPIN8* have two paralog genes (*PaPIN2a* and *PaPIN2b*) and (*PaPIN8*a and *PaPIN8b*), respectively, while PaPIN5 only has one. The PIN genes can also be classified according to Bennett et al. [24], as follows: eight canonical PIN genes (*PaPIN1a-PaPIN1e*, *PaPIN2a*, *PaPIN2b*, and *PaPIN3*), three non-canonical (*PaPIN5*, *PaPIN8a*, and *PaPIN8b*), and one intermediate (*PaPIN6*).

The avocado genome only has the canonical *PINs PIN1*, *PIN2*, and *PIN3*, the non-canonical *PINs PIN5* and *PIN8*, and the intermediate *PIN6*. Compared to Arabidopsis, the PIN4 and PIN7 genes were not found in this study. Similar results have been reported in maize [89], cotton [46], soybean [27], ginseng [32], and rice [25]. In maize, orthologs of AtPIN2, AtPIN4 and AtPIN7 are not present in the genome [89]. However, four PIN genes (ZmPIN1-ZmPINd) are present in maize. Studies in maize have suggested that the ZmPIN1 genes have functional redundancy [50], and although PIN4 and PIN7 are not present in maize, it has been suggested that the PIN1 genes could have acquired a certain degree of subfunctionalization, and therefore ZmPIN1 could perform the same function as the genes PIN4 and PIN7 [44]. In Arabidopsis, single mutants showed defects in the early stages of the embryo; however, they recovered and managed to re-establish the axis, while quadruple mutants failed to establish apical–basal polarity. These results demonstrated a functional redundancy among the PIN genes [57,90]. In addition, it was suggested that ectopic expression of PIN genes could compensate for the function of the missing PIN genes [87,90]. It is necessary to know more about each PIN transporter’s specific function to determine what each one’s absence means. The presence of a family of genes for *PIN* is possibly related to the importance of this transporter in the polar movement of the IAA; a few species have only one gene for *PIN1*, examples being *A. thaliana* [88] and *S*. *lycopersicum* [26].

Most studies that analyze the PIN protein sequence show high conservation along the biological scale [23,40,91]. The results shown in Figure 1 clearly illustrate that there is a clear phylogenetic relationship between the PaPIN proteins with the sequences of the PIN proteins of *A. thaliana*, *O. sativa*, and *S. lycopersicum*. We found 92% homology between the PaPIN6 proteins with SlPIN6, while PaPIN5-AtPIN5 and PaPIN3-SlPIN3 only had 54 and 53% homology, respectively. In Arabidopsis, the identity between two family members varies from 32% in the case of AtPIN5-AtPIN8 to 85% in the case of AtPIN3-AtPIN7. When compared to their bacterial counterparts, these relatively high values suggest that all PIN genes in higher plants diverged from a single ancestral sequence.

All PIN proteins have a central hydrophilic loop of varying length, flanked by several, mostly conserved, N- and C-terminal transmembrane domains. The canonical genes (PIN1–PIN4, and PIN7) have a structure with their long cytoplasmic loop that contains regulatory elements that are absent from the short PINs [9] and are involved in different aspects of plant physiology, and the non-canonical ones (PINs PIN5 and PIN8) are involved in the lattice in homeostasis. The PIN6 transporter has only a subset of the regulatory elements [24].

The gene structure analysis showed nine PaPIN genes contained six exons and five introns, except for PaPIN2b, PaPIN5, and PaPIN6 [27,31,45,92]. The number of introns is very similar to that of rice. This species has four to six introns [25], and *Phyllostachys edulis* has an average of five introns [49]. However, exon–intron structure tended to be conserved among the PaPIN genes, which was similar to that reported in other plants, suggesting that intron–exon organization of PIN genes is highly conserved [15].

The predicted transmembrane helices of the PaPIN proteins showed a conservative structure, as in Arabidopsis and *O. sativa* [25]. The average length of the PaPIN proteins is 606 amino acids for canonical PINs, while that of the non-canonical PINs is 398 amino acids, and that of the intermediate PINs is 509 amino acids. The size of the PIN proteins in Arabidopsis varies between 351 (AtPIN5) and 647 (AtPIN2) amino acids in length [88]. These differences are accentuated when the lengths of the intermediate zones are analyzed. The average length of the central hydrophilic loop was 341 amino acids for long PaPINs. On the other hand, for short PaPINs, the length of the central hydrophilic loop was 50, 168, and 134 amino acids for PaPIN5, PaPaPIN8a, and PaPIN8b, respectively. These values in the lengths of the genes and their components are among the values reported for other species. These data and phylogenetic trees suggest that all PIN genes in higher plants come from a common ancestor [15].

Given the high identity of the genes that code for PIN proteins among higher plants, it is unsurprising that motifs are conserved among PIN protein homologs. This, in turn, leads to, for example, all the canonical PIN proteins being located in the plasmalemma and mediating the intercellular transport of the IAA.

On the other hand, the difference in the length of the PIN genes means the proteins they codified will have more or fewer motives and different structures, leading to their localization in other membranes and possibly different functions. For example, it is interesting that PaPIN1a, which does not have only nine motifs, localizes in the chloroplast membrane, while PaPIN5, a non-canonical PIN, localizes in the vacuole membrane (Table 1). Ganguly et al. [93] determined that non-canonical PIN proteins have shorter hydrophilic loops and are located in the endoplasmic reticulum. In our case, PaPIN5 is located in the tonoplast. This fact is relevant, since plant cells accumulate a large number of IAA conjugates in the vacuole, and these can serve as a source of free IAA [94,95]. The vacuole will need to have a system to transport the IAA to the cytoplasm. PIN5 may carry out this role. The non-canonical genes (PaPIN8a and PaPIN8b) and the intermediary gene (PIN6) localize in the plasmalemma. They may also localize in the endoplasmic reticulum, as suggested for Arabidopsis [96]. It is important to note that PAPIN5 does not have the phosphorylation sites characteristic of the other PIN transporters, such as S209, S210, and S248, as well as the Y302 and T252 sites (Figure 8). This absence of phosphorylation could suggest that its location is fixed in the tonoplast, since it has been suggested that phosphorylation provides mobility to the PIN transporters [97,98].

Auxins are essential in the formation of embryos, both somatic and zygotic [75,99]. We have also previously found a burst in the expression of genes related to auxin homeostasis during somatic embryogenesis in *Coffea canephora* [100]. Therefore, it is not surprising that their transporters have high expression levels in most cases, and that only a few are more discreet in their expression (Figure 9). We determined that during the zygotic embryogenesis of *P. americana*, the 12 genes found in its genome are expressed (Figure 5). Six *PIN* genes belonging to group I (*PaPIN1a*, *PaPIN1b*, *PaPIN1d*, *PaPIN1e*, *PaPIN2b*, and *PaPIN6*) showed high expression levels during the first weeks of zygotic embryo formation. Among the group I genes, PaPIN1a showed the highest expression level during the evaluated stages. Except for PaPIN1c, all copies of PIN1 found in this study showed a high expression level all through embryo development, suggesting that PIN1 is required during the formation and development of the zygotic embryo in avocado. The expression level of PaPIN2a was lower, compared to PaPIN2b. The lowest expression level was observed in PIN8a, PIN8b and PIN5. These results suggested that *PaPIN1a*, *PaPIN2b*, *PaPIN1d*, *PaPIN6*, *PaPIN1*b, and *PaPIN1e* are required in the early stages of embryo development of avocado. This expression of the *PIN* genes has also been observed during the SE of other species, such as *Lilium pumilum* [101], in which eleven PIN/PILS family transcripts were determined; in *Carica papaya*, *CpPIN1*, *CpPIN3*, and *CpPIN4* were expressed during the development of the somatic embryo [102].

Different groups working on the zygotic embryogenesis of *A. thaliana* have determined the importance of PIN genes for this process to be carried out correctly [57,103,104,105,106]. Through immunolocalization studies and markers such as the green fluorescent protein [57,107,108], the importance of the PIN1, PIN4, and PIN7 transporters, as well as the ABCB1 and ABCB19 transporters, have been determined [57,87]. It has been established that PIN7 is involved in the establishment of the auxin gradient during the preglobular stage. After fertilization, the first asymmetric division generates the apical cell and the cells that will give rise to the suspensor. In this last cell, IAA is produced, which is transported to the apical cell through PIN7. Once the apical cell divides for the first time, PIN1 is the transporter responsible for mobilizing the IAA between the parts. When the globular stage is reached, there is a rearrangement of the PIN7 transporters, and they pass to the basal part of the suspensor cells with the support of PIN4. They reverse the flow of the IAA, accumulating it in the forming hypophysis [77].

The 3D structures of the twelve members of the PaPIN family of *P. americana* (Figure 5a,c,d and Figure 6a–l) showed the characteristic structure of the PIN transporters [24], including the presence of four amino acids essentials for the binding of the IAA (V46, N112, N548, and I675). The predicted structure and topology of the canonical PaPIN1s are similar to the structures of the canonical AtPIN1 and AtPIN3 recently reported [6,109]. On the other hand, Ung et al. [17] elucidate the structure for AtPIN8, a non-canonical PIN in the presence and absence of IAA. Our data for the predicted structure of both PaPIN8 types show the same basic architecture (Figure 6).

It has been documented that polarity and transport activity are regulated by phosphorylation through several protein kinases [110,111,112]; the target for the kinases is the region that corresponds to the cytosolic loop or hydrophilic loop (Figure 5a and Figure 6a–l) [113]. This protein modification is central for the polar location of the PINs in the plasma membrane [49,110,111]. We found that Ser210 is a conserved amino acid among PIN proteins and is phosphorylated in *A. thaliana* [97]. In the case of PaPIN6, this residue (S209) is also present, and residues 248 (threonine in *A. thaliana* and serine in *P. americana*) and 252 (serine in *A. thaliana* and threonine in *P. americana*) are also phosphorylated. The amino acid is changed, but the phosphorylation is not; thus, the potential function of phosphorylation is preserved.

## 4. Materials and Methods

### 4.1. Phylogenetic Tree Construction

The PIN protein sequences from *A. thaliana*, *O. sativa*, *S. lycopersicum* and *P. americana* were retrieved from the TAIR website (https://www.arabidopsis.org/, accessed on 5 October 2022), the Rice Genome Annotation Project website (http://rice.uga.edu/index.shtml, accessed on 6 October 2022), the International Tomato Genome Sequencing Project (https://solgenomics.net/organism/Solanum_lycopersicum/genome, accessed on 8 October 2022), and the CoGe website (https://genomevolution.org/coge/SearchResults.pl?s=29302&p=genome, accessed on 14 September 2022), respectively. The full-length amino acid sequences were aligned using ClustalW, with the default parameters. Then, the resulting alignment was used to construct a phylogenetic tree with the neighbor joining method [114]. Poisson distances, pairwise deletion, and 1000 bootstrap replications using the MEGA11 software were carried out [115].

### 4.2. Identification and Theoretical Characterization of the PaPIN Protein Family in Avocado

The physicochemical characteristics of PIN proteins of avocado were analyzed using the ExPASy ProtParam tool (https://web.expasy.org/protparam/, accessed on 27 September 2022), the prediction of the transmembrane helices of PIN proteins was performed using TMHHMv.2.0 [116] (https://services.healthtech.dtu.dk/service.php?TMHMM-2.0, accessed on 28 September 2022), and the subcellular protein localization was predicted using WoLF PSORT (https://www.genscript.com/wolf-psort.html, accessed on 28 September 2022).

### 4.3. Gene Structure and Motif Analysis

The analysis was performed on the Gene Structure Display Server (GSDS) (http://gsds.gao-lab.org/, accessed on 17 October 2022). The coding sequences with their corresponding genomic sequences of the PIN genes of avocado were used to identify the CDS-intron structure. The conserved motifs were identified using Multiple EM for Motif Elicitation (MEME) (https://meme-suite.org/meme/, accessed on 17 October 2022). The maximum number of motifs was 10.

### 4.4. Plant Material, RNA Extraction and Transcriptome Analysis

Fruits of eight different sizes (1 cm: ZE_1; 2 cm: ZE_2; 3 cm: ZE_3; 4 cm ZE_4; 5 cm: ZE_5; 7 cm: ZE_7; 8 cm: ZE_8 and 9 cm: ZE_9) of P. americana Mill cv. Hass were collected in Uruapan, Michoacán, Mexico, and from orchards, during two different blossom seasons. We used fruit size rather than time after flowering, due to the impossibility of controlling pollination in avocado trees. This procedure was used due to the number of flowers on the tree and the fact that the flowers of different sexes open at different times.

Total RNA was isolated from 100 mg zygotic embryos of each fruit size, following the protocol reported by Djami-Tchatchou and Straker [117]. RNA concentration was measured at 260/280 nm using NanoDropTM 2000 (Thermo Fisher Scientific, San Jose, CA, USA), and RNA integrity was assessed by agarose gel electrophoresis. RNA was sequenced on the Illumina NextSeq 500 platform in paired-end mode (Novogene Corporation Inc. 2921, Stockton Blvd., Suite 1810. Sacramento CA 95817, USA). Three independent biological replicates of each fruit size were processed. The quality of the reads was verified using FastQC (http://www.bioinformatics.babraham.ac.uk/projects/fastqc/ (accessed on 16 July 2022)); then, the reads were trimmed and filtered with Cutadapt [118], with default parameters. An index of the reference genome [85] was built using Bowtie2-build, and then paired-end reads were aligned with the reference genome, using Bowtie2 [119]. The count of the reads aligned with each gene was performed with the HTSeq-count (v. 0.10.0) [120], with the default parameters. To prove the similarity between the biological replicates, we calculated Pearson’s correlation coefficient using the quartile normalization method, and the normalized data were transformed to Log2 (x + 1) using the Rstudio software (v. 1.1.456). Differential expression analysis was performed using the DEGSeq2 (v1.21.0) [121] R package with an adjusted *p*-value of 0.05, and log2 of 1.5 (LFC ≤ 1.5 or LFC ≥ 1.5) was set as the threshold for significant differential expression. Heat maps were generated in the ggplot2 package for R.

### 4.5. Building of 3D Structures, Molecular Modelling, Multiple-Sequence Alignment and Prediction of Transmembrane Segments of PaPIN1 Proteins from Persea americana

The amino acid sequence was used to predict the building of the PaPIN auxin transport proteins. The 3D structures were built using the SWISS-MODEL software, accessible via the Ex-PASy web server (https://swissmodel.expasy.org//; accessed on 27 September 2022). The best-predicted models were evaluated using the global model quality estimation (GMQE) and assessed after model building using the QMEAN global score. The IAA 3D structure was built from the molecular formula using the structure edition tool with the build structure option of the UCSF Chimera 1.14 software [122]. The molecular formula was downloaded from PubChem (https://pubchem.ncbi.nlm.nih.gov//; accessed on 27 September 2022). The UCSF Chimera 1.14 Molecular Graphics Systems was used to model and visualize the 3D structures [122]. The TMhelix server was used to predict the transmembrane segments: https://dtu.biolib.com/DeepTMHMM version 1.0.13 (accessed on 24 October 2022). The multiple sequence alignment was carried out with MUSCLE (MUltiple Sequence Comparison by Log-Expectation) at https://www.ebi.ac.uk/Tools/msa/muscle/ (accessed on 4 October 2022) (EMBL-EB, Hinxton, UK) software, using default parameters. The amino acid sequence of the PaPINs was used for the alignment, and AtPIN1 (code PDB: 7Y9V) was used as the reference protein.

### 4.6. Prediction of the Phosphorylation Sites

The amino acid sequences of each PaPIN protein were used to predict the phosphorylation sites with the NetPhos-3.1 online server (https://services.healthtech.dtu.dk/services/NetPhos-3.1/, accessed on 26 April 2023).

## 5. Conclusions

Auxins are involved in a large part of the plant development process. It is a growth regulator that moves to exert its action, and it does so over long distances and from cell to cell. Much of that transport requires specialized molecules, the PIN transporters. These transporters can be grouped into three classes depending on their size: the canonical (the largest), the non-canonical (the smallest), and the intermediate ones. In the case of *P. americana*, we determined that the three classes exist and that the predicted structures corresponded to the classes in which they were placed. The PaPIN proteins share phosphorylation sites with other PIN proteins, and their expression pattern during the development of the zygotic embryo will help with understanding the problem of the SE process in the avocado.

The presence and function of PIN transporters have not been analyzed in the required detail. This area should be studied in detail, since the response to auxins depends on its homeostasis, which in turn depends on the transport of auxin from its sites of synthesis. This transport is fascinating in the case of zygotic embryogenesis, since the accumulation of auxins in specific cells initiates the differentiation process of those cells.

In the case of *P. americana*, the next step will be to determine the location of each PIN transporter in the membrane of the developing zygotic embryo and the sites of accumulation and action of IAA.

## Figures and Tables

**Table 1:** Members of the PaPIN family are found in the avocado genome.

			Deducted Polypeptide					
Gene	ORF Length (bp)	No. of Exons	Length (aa)	Molecular Weight (Da)	pI	GRAVY	No. of Trans-Membrane	Subcellular Localization
PaPIN1a	1710	6	569	61,971.01	8.66	0.226	9	Chloroplast
PaPIN1b	1887	6	628	68,306.87	8.10	0.198	9	Plasma membrane
PaPIN1c	1791	6	596	64,915.32	9.09	0.226	8	Plasma membrane
PaPIN1d	1917	6	638	69,598.27	8.85	0.102	9	Plasma membrane
PaPIN1e	1917	6	638	69,598.27	8.85	0.102	9	Plasma membrane
PaPIN2a	1983	6	660	71,911.03	9.45	0.086	8	Plasma membrane
PaPIN2b	1599	5	532	58,012.56	6.57	0.111	5	Plasma membrane
PaPIN3	1767	6	588	63,460.40	9.24	0.137	8	Plasma membrane
PaPIN5	1074	5	357	39,164.18	6.95	0.722	9	Vacuolar
PaPIN6	1530	7	509	55,370.96	8.91	0.410	9	Plasma membrane
PaPIN8a	1311	6	436	48,444.16	9.12	0.404	8	Plasma membrane
PaPIN8b	1209	6	402	44,630.07	9.43	0.529	8	Plasma membrane

## Data Availability

Not applicable.
